# Desorption Electrospray Ionization (DESI) Mass Spectrometric Imaging of the Distribution of Rohitukine in the Seedling of *Dysoxylum binectariferum* Hook. F

**DOI:** 10.1371/journal.pone.0158099

**Published:** 2016-06-30

**Authors:** Patel Mohana Kumara, Amitava Srimany, Suganya Arunan, Gudasalamani Ravikanth, Ramanan Uma Shaanker, Thalappil Pradeep

**Affiliations:** 1 DST Unit of Nanoscience and Thematic Unit of Excellence, Department of Chemistry, Indian Institute of Technology Madras, Chennai, 600036, India; 2 School of Ecology and Conservation, Department of Crop Physiology, University of Agricultural Sciences, GKVK, Bengaluru, 560065, India; 3 Ashoka Trust for Research in Ecology and the Environment, Royal Enclave, Sriramapura, Jakkur, Bengaluru, 560064, India; University of San Agustin, PHILIPPINES

## Abstract

Ambient ionization mass spectrometric imaging of all parts of the seedling of *Dysoxylum binectariferum* Hook. f (Meliaceae) was performed to reconstruct the molecular distribution of rohitukine (Rh) and related compounds. The species accumulates Rh, a prominent chromone alkaloid, in its seeds, fruits, and stem bark. Rh possesses anti-inflammatory, anti-cancer, and immuno-modulatory properties. Desorption electrospray ionization mass spectrometry imaging (DESI MSI) and electrospray ionization (ESI) tandem mass spectrometry (MS/MS) analysis detected Rh as well as its glycosylated, acetylated, oxidized, and methoxylated analogues. Rh was predominantly distributed in the main roots, collar region of the stem, and young leaves. In the stem and roots, Rh was primarily restricted to the cortex region. The identities of the metabolites were assigned based on both the fragmentation patterns and exact mass analyses. We discuss these results, with specific reference to the possible pathways of Rh biosynthesis and translocation during seedling development in *D*. *binectariferum*.

## Introduction

Chromone alkaloids consist of a noreugenin chromone (5,7-dihydroxy-2-methylchromone) component linked with a ring containing one or more nitrogen atoms [1–3]. These metabolites are structurally diverse and are derived from the convergence of multiple biosynthetic pathways that are widely distributed in plant (Meliaceae and Rubiaceae) and animal kingdoms [2, 4–8]. The natural occurrence of rohitukine (Rh), a chromone alkaloid, is restricted to only five plant species; *Amoora rohituka* [4], *Dysoxylum binectariferum* [5], *D*. *acutangulum* [7] (all from the Meliaceae family), *Schumanniophyton magnificum*, and *S*. *problematicum* [1, 2] (from the Rubiaceae family). Among these species, *D*. *binectariferum* accumulates the highest amount of Rh (3–7% by dry weight in stem bark) [9, 10]. Recently, dysoline, a new regioisomer of Rh was reported from the stem bark of *D*. *binectariferum* [11]. Besides the plant sources, endophytic fungi associated with *A*. *rohituka* and *D*. *binectariferum* have also been shown to produce Rh in culture, independent of the host tissue [12–14]. The biosynthetic pathway of chromone alkaloids in general, and Rh in particular, have not been elucidated [15], though the occurrence of free noreugenin in the plant suggests that it might be formed prior to the conjugation with the nitrogen-containing moiety. The presence of trigonelline in chromone alkaloid producing plants suggest that it could be a possible precursor for pyridine-related alkaloids [1, 2]. Recent studies have reported a pentaketide chromone synthase (PCS) that catalyzes the formation of the noreugenin compound, 5,7-dihydroxy-2-methylchromone, from five malonyl-CoA precursor units [16–19]. Studies have also reported that ornithine is an initial precursor molecule for the biosynthesis of piperidine, nicotine, and tropane alkaloids [20]. The nitrogenous group derived from ornithine could be the origin of the nitrogen atom in chromone alkaloid biosynthesis.

Pharmacologically, Rh has been reported to have anti-inflammatory, anti-fertility, anti-implantation, anti-cancer, and anti-adipogenic activities besides having immuno-modulatory properties [2, 4–8, 11, 21]. Two derivatives, namely, flavopiridol (also known as HMR 1275 or alvocidib) and P-276-00 have been shown to competitively bind to the ATP binding pocket of cyclin-dependent kinases (CDKs) and inhibiting their activity. Flavopiridol arrests the cell cycle at both G1 and G2 phases and has been shown to be effective against breast and lung cancers and chronic lymphocytic leukemia [22, 23]. The compound has been approved as an orphan drug for treatment of chronic lymphocytic leukaemia [8]. Flavopiridol has also been shown to block human immuno-deficiency virus Tat trans-activation and viral replication through inhibition of positive transcription elongation factor b (P-TEFb) [24, 25]. The derivative P-276-00 is in phase II clinical studies for advanced refractory neoplasms and multiple myeloma [8].

In a recent study, we have examined the spatial and temporal distribution pattern of Rh and related compounds in different parts of the seeds of *D*. *binectariferum* [26]. Rh (*m/z* 306.2) accumulation increased from early seed development to seed maturity and was largely found in the embryo and cotyledon. Besides Rh, we also reported the presence of Rh acetate (*m/z* 348.2), and glycosylated Rh (*m/z* 468.2) in the seeds.

In this study, we examine the spatial distribution of Rh and related compounds in seedlings of *D*. *binectariferum* using desorption electrospray ionization mass spectrometry imaging (DESI MSI). In DESI MS, molecular masses are analyzed by transporting desorbed ions generated by spraying electrically charged solvent droplets at the sample of interest into the mass spectrometer [27]. This is an ambient ionization technique and consequently many of the limitations of conventional mass spectral analysis do not apply here. In recent years, the technique has been used widely to spatially map the occurrence of a number of plant secondary metabolites and infer the underlying mechanisms leading to spatial patterns as well as their adaptive significance [28–33]. MALDI MS has been used to identify metabolites in glandular trichomes from a wild tomato (*Solanum habrochaites*) leaf at a spatial resolution of around 50 μm [34]. More recently, mass spectrometric imaging have been used in conjunction with tissue specific transcriptomic analysis to deduce the biosynthetic pathway; for example, in Flax (*Linum usitatissimum*) [35], *Arabidopsis* [36], and *Podophyllum* species [37, 38]. The increasing use of MS imaging for spatial pattern analysis owes itself to its relative ease of use and its unique advantage, especially when detecting relatively labile compounds that may lose their structural and chemical characteristics upon extraction.

In the present study, using DESI MSI we have mapped the spatial distribution of Rh and other related compounds in the seedlings of *D*. *binectariferum*. The identities of most of the metabolites, including Rh were assigned based on both the fragmentation patterns and exact mass analyses. We discuss these results, with specific reference to the possible pathways of Rh biosynthesis and translocation.

## Materials and Methods

### Ethics statement

The fieldwork and collection of seed sample of *D*. *binectariferum* was carried out in the central Western Ghats regions of Karnataka (Jog, 14^0^ 13’ 65” N, 74^0^ 48’ 35” E) with kind permission from the Karnataka Forest Department, Bengaluru. Seed sampling was carried out under the supervision of forest officers and used solely for scientific research. The sampling was non-invasive with no impact on the natural growth or regeneration of *D*. *binectariferum* populations in the wild.

### Plant material

*D*. *binectariferum* (diploid chromosome number 2n = 80) is a medium to large sized tree distributed in the tropical and subtropical regions of Eastern Himalayas, Khasi Hills, Western Ghats of Peninsular India, and Sri Lanka. In the Western Ghats, *D*. *binectariferum* is distributed from Coorg to the Anamalais and Tinnevelly in the moist forest [39]. The plant is pollinated by insects [40] while the seeds are dispersed by birds (hornbills and imperial pigeons). Seedling recruitment in the natural habitats is limited because of heavy predation of fruits by rodents [41].

*D*. *binectariferum* seeds were collected from Jog (14^0^ 13’ 65” N, 74^0^ 48’ 35” E) located in the central Western Ghats, India. From these seeds, seedlings were raised in polybags in the Forest Nursery at the University of Agricultural Sciences, GKVK, Bengaluru and later transported to the Indian Institute of Technology Madras campus at Chennai. Seedlings were maintained under shade and were well watered. Root, shoot, leaves, and cotyledons of 10 month old seedlings were used for the imaging studies.

### Extraction of Rh and HPLC analysis

**Fig 1:**
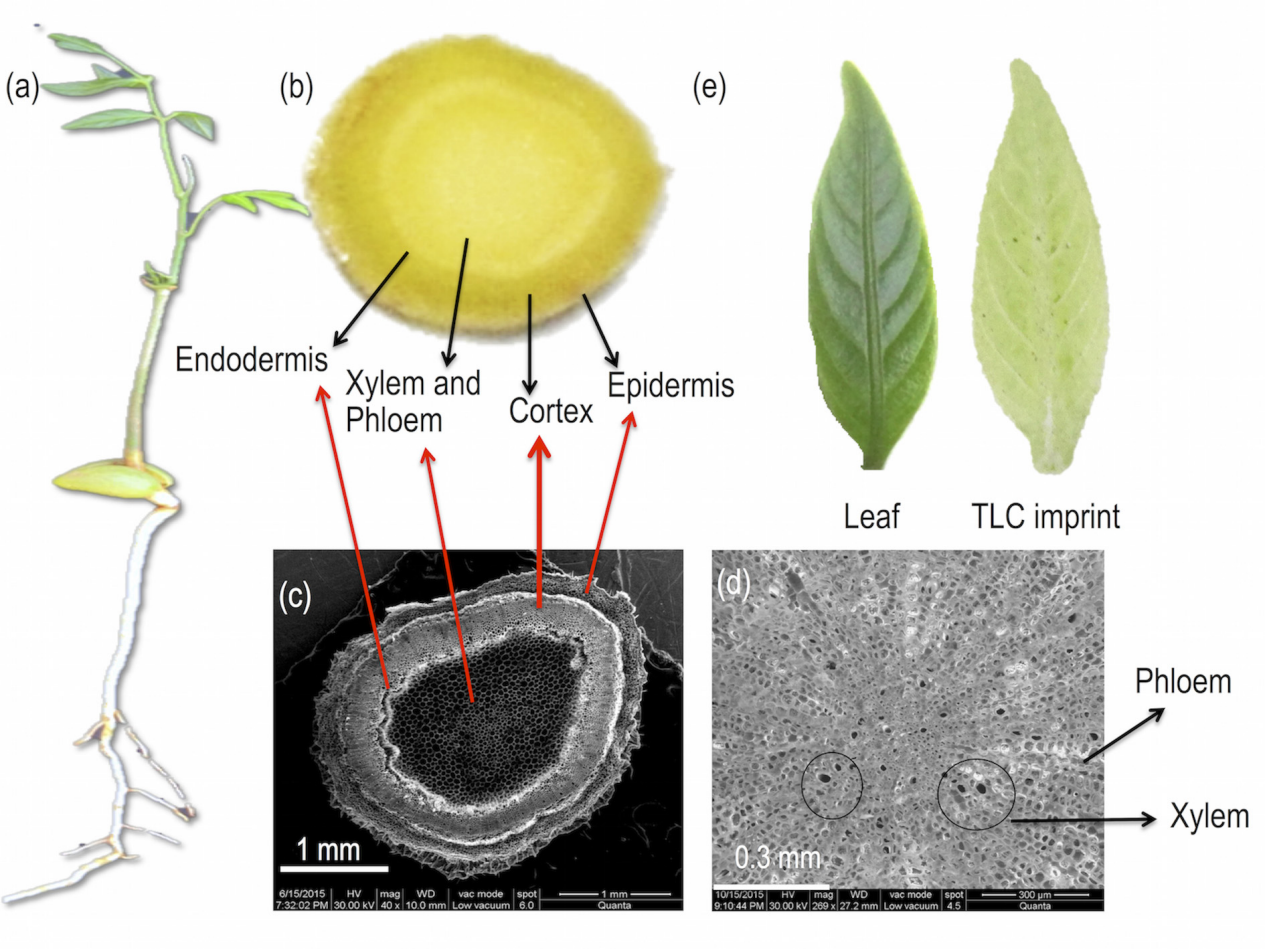
(a) 10 month old seedling of *D*. *binectariferum*, (b) Cross section of the root, (c) Scanning electron microscope (SEM) image of cross section of the root, (d) Magnified SEM image of the root showing the xylem and phloem, and (e) Leaf and its TLC imprint.

10 months old seedlings (n = 3), about 25 cm in length (Fig 1A), were collected from the nursery and oven dried for 4 days at 70°C. Rh was extracted from different parts of the seedlings (root, stem, and leaves) following earlier report [9]. Rh content (w/w) in the extracted samples were analyzed using reverse-phase HPLC (Shimadzu, LC20AT, Japan), RP-18 column (4.6 X 250 mm, 5 μm) with UV absorbance at 254 nm [9, 26]. The solvent system comprised of acetonitrile and 0.1% TFA as mobile phase. A gradient starting from 0%:100% to 100%:0% of acetonitrile:0.1% TFA with a flow rate of 1000 μL/min was used. Sharp peak with highest peak intensity was obtained at a mobile phase composition of 30% acetonitrile and 70% TFA (0.1%). All samples were then analyzed in an isocratic mode using 30% acetonitrile:70% TFA (0.1%) as mobile phase. A standard curve was constructed using various concentrations (0.125, 0.25, 0.5, 0.75, and 1 mg/mL) of Rh [9]. 20 μL of each of the standard concentrations were injected into the HPLC and their respective retention times (R_t_) and peak areas were recorded. The peak area was plotted against the respective concentration and a regression analysis was carried out. Using the regression equation, Rh content was calculated per 100 g dry weight basis. All estimates were made over 3 replicate samples.

### ESI MS analysis

Different sections of the root, shoot, leaves, and cotyledons (corresponding to regions that were used for the imaging) were cut into small pieces and soaked in methanol for 12 hours. The solution was filtered and centrifuged at 10000 rpm for 10 minutes. The supernatant was analyzed by ESI MS/MS using Thermo Scientific LTQ XL (Thermo Scientific, San Jose, CA, USA) mass spectrometer and exact mass was analyzed using Thermo Scientific Orbitrap Elite (Thermo Scientific, San Jose, CA, USA) mass spectrometer. The data was acquired in positive ion mode with a spray voltage of 5 kV. Collision induced dissociation (CID) was used for fragmentation of the ions during MS/MS measurements. The identities of the ions were established based on both the fragmentation patterns and exact masses of the ions obtained and using METLIN metabolite database [42]. The mass tolerance of ±3 ppm was used in the METLIN database search. The MS/MS data was used to infer the compound identity by comparing the fragment ion *m/z* with published literature and database. All the spectra are represented in the profile mode.

### Desorption electrospray ionization mass spectrometry imaging (DESI MSI)

Using a surgical blade, seedlings (10 months old) were neatly cut to separate the root, stem, and meristem. Longitudinal and cross sections of different plant parts were made according to the experimental needs. Cross sections (about 2 mm thick) were made at every 1 cm interval from the root tip to the shoot tip while longitudinal sections were made approximately at the median axis of the root and stem. Cross section of the root (Fig 1B) was observed under FEI Quanta 200 environmental scanning electron microscope (ESEM). The images clearly showed the parts corresponding to the epidermis, endodermis, cortex, xylem, and phloem tissues (Fig 1C and 1D).

To get an imprint of the molecules present on the cut-end of the section on a flat surface, a TLC plate (TLC Silica gel 60 F_254_, Merck KGaA, Germany) was pre-wetted with methanol and kept on a heating mantle (~70°C). The cut sections (both cross and longitudinal sections) were placed on the hot TLC plate and hand pressed for 10 seconds to get an imprint [26, 43]. Leaves of 10 months old seedlings were imprinted on a TLC plate (Fig 1E) with 2 ton pressure for 15 to 30 seconds using a hydraulic pelletizer [32].

Imaging experiments were conducted using Thermo Scientific LTQ XL (Thermo Scientific, San Jose, CA, USA) mass spectrometer with 2D DESI ion source (Omni Spray Ion Source) from Prosolia, Inc., Indianapolis, IN, USA. The DESI source conditions were as follows; nebulizing gas (dry nitrogen) pressure: 150 psi, spray angle: 60° to the sample surface, tip of spray to sample surface distance: 1 mm, tip of spray to mass spectrometer inlet distance: 3 mm, spray solvent: methanol, solvent flow rate: 5 μL/min, spray voltage: 5 kV, and ionization mode: positive (+ve). The image area was chosen according to the sample dimensions and the spatial resolution used was 250 μm X 250 μm. Imaging 1 cm X 1 cm area of tissue sample took approximately 30 min. Imaging time varied with the area of the tissue samples. For a seedling of 25 cm in length, it took about 12 hours to completely image the whole seedling. Image files (IMG File) were created using FireFly software from the acquired data and BioMAP software were used to process the image files to create images. Normaliztion of the images were done for the individual ions in a particular Figure. While processing the image files in BioMAP, separate images could be generated with a difference of 0.083 in *m/z*. The *m/z* values obtained from BioMAP image had been approximated to one decimal place. For example, in case of Rh (*m/z* 306.2, obtained from DESI MS data), we generated image in BioMAP at *m/z* 306.167 instead of *m/z* 306.250, as the latter deviated more from the experimental value. In this process, we might encounter a situation where, for a particular ion, images may actually represent several species, as biological samples are complex mixtures. However, Orbitrap data excluded the possibility of existence of other ions in close proximity to the ions of interest, although a single *m/z* value might represent different isomers.

## Results and Discussion

### Metabolite identification via ESI MS/MS and exact mass measurement

**Fig 2:**
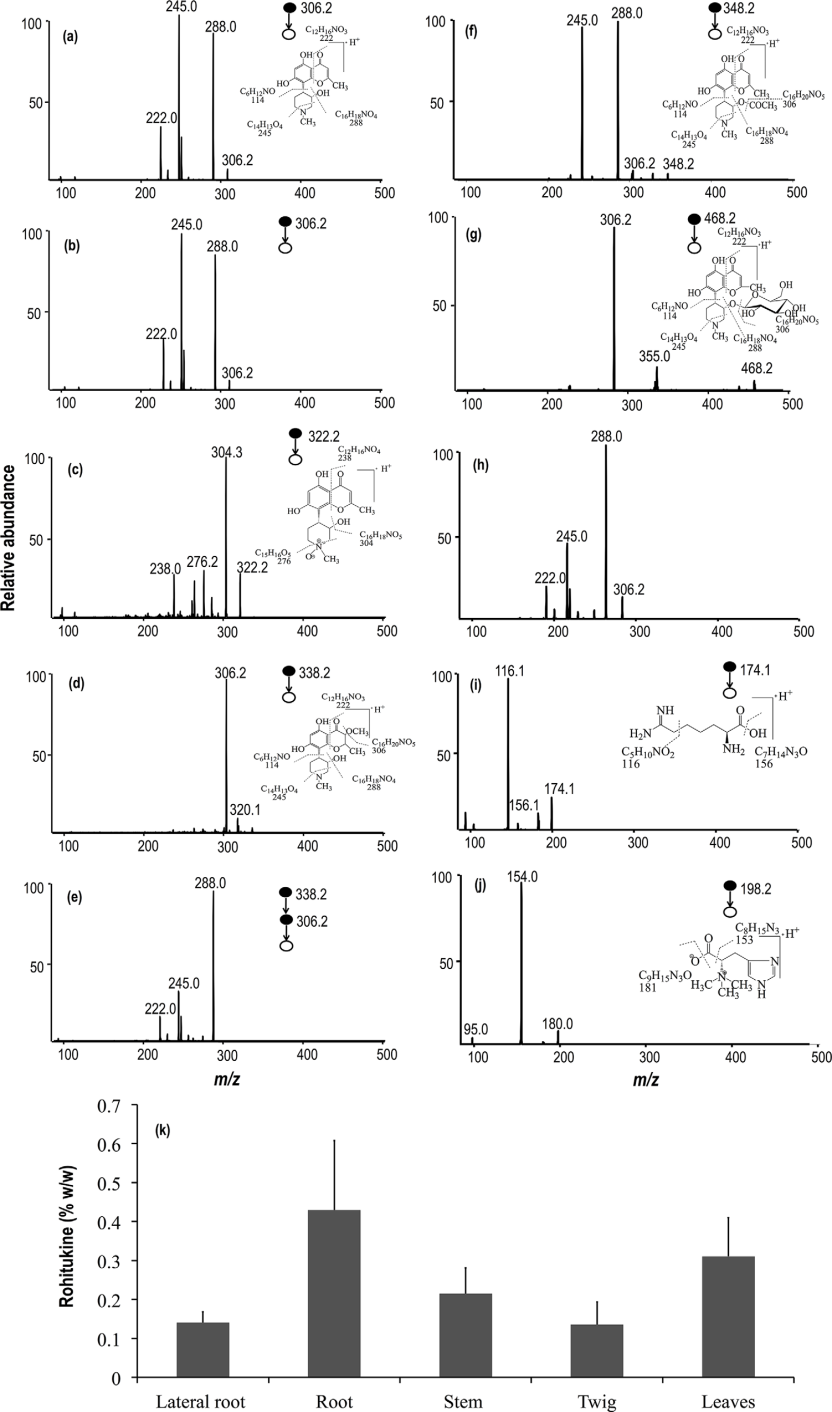
ESI MS/MS fragmentation patterns of (a) standard Rh, (b) plant Rh, (c) Rh-N-oxide, (d, e) methoxylated Rh, (f) Rh acetate, (g, h) glycosylated Rh, (i) indospicine, and (j) hercynine. Insets in images show the schemes of mass fragmentation of the respective metabolites. (k) Rohitukine content (% w/w) in different parts of 10 month old seedlings of *D*. *binectariferum*.

ESI MS analysis of the seedlings showed molecular signatures in the range of *m/z* 140–1000 (S1 Table). These masses included those of Rh, its related compounds and several unknowns. For the purpose of this paper, we restrict our further analysis to only the chromone alkaloids and their possible precursors. The ions were subjected to fragmentation using CID during ESI MS/MS analysis (Fig 2 and S1 Table). Considering the fragmentation patterns and the exact masses of the ions, probable chemical formulae and structures of the chromone alkaloids and their precursors were arrived at using the METLIN metabolite database (Fig 2) [42]. A list of *m/z* values of the parent and fragment ions, their probable chemical formulae, and structures are given in S1 Table.

#### Rh and related chromone alkaloids

The parent molecule Rh (*m/z* 306.2) fragmented into daughter ions at *m/z* 288.0, 245.0, and 222.0 (Fig 2B) similar to the daughter ions obtained from a standard (Fig 2A). The peak at *m/z* 288.0 is due to the neutral loss of H_2_O from the piperidine ring and *m/z* 245.0 is due to further fragmentation of the ring. The peak at *m/z* 222.0 is due to the fragmentation of the chromone ring [14, 44].

Rh-N-oxide (*m/z* 322.2) was recovered from leaves. Fragmentation of this molecule yielded three ions at *m/z* 304.3, 276.2, and 238.0. The peak at *m/z* 304.3 is due to the neutral loss of H_2_O from the piperidine ring and *m/z* 276.2 is due to further fragmentation of the piperidine ring. The peak at *m/z* 238.0 is due to the fragmentation of the chromone ring (Fig 2C) [14].

Beside these, we also obtained an ion at *m/z* 338.2 from leaves, which is most likely, a methoxylated analouge of Rh. The MS/MS fragmentation of *m/z* 338.2 yielded *m/z* 306.2 which could be accounted for by the neutral loss of methanol (CH_3_OH) (Fig 2D and 2E). The ion at *m/z* 306.2 upon further fragmentation yielded three major ions at *m/z* 288.0, 245.0, and 222.0 similar to that of Rh. The peak at *m/z* 288.0 is due to the neutral loss of H_2_O from the piperidine ring and *m/z* 245.0 is due to further fragmentation of the ring (Fig 2E). Two other chromone alkaloids, namely, Rh acetate (*m/z* 348.2) (Fig 2F), and glycosylated Rh (*m/z* 468.2) were also recovered from the seedlings (Fig 2G and 2H).

Two other ions, *m/z* 328.2 and 610.9, were predicted to be sodiated Rh and protonated dimer of Rh, respectively. Their exact masses, *m/z* 328.1155 and 611.2598, indicated probable chemical formulae of C_16_H_19_NO_5_Na and C_32_H_39_N_2_O_10_, respectively (S1 Table) [26]. A summary of the fragmentations obtained is presented in S1 Table.

#### Probable precursors of chromone alkaloids

Besides Rh and its analogues, a few small molecules with *m/z* 174.1 and 198.2 were observed in the seedlings of *D*. *binectariferum*. Fragmentation patterns of these ions are shown in Fig 2I and 2J. Exact *m/z* of these compounds measured by Orbitrap at 174.1236 and 198.1235 indicated that they could be indospicine (C_7_H_15_N_3_O_2_) and hercynine (C_9_H_15_N_3_O_2_), respectively (S1 Table). Interestingly, both of these molecules are reported in KEGG metabolic pathway database to serve as precursors (acyl group in the cyclization) in the biosynthesis of piperidine and pyridine alkaloids and also in the biosynthesis of various fatty acids. It is likely that these molecules may also be involved in the biosynthesis of chromone alkaloids in seedlings of *D*. *binectariferum*.

### Rh quantification via HPLC

Among the chromone alkaloids, Rh was predominant in all tissues examined. Rh content was highest in the main roots (0.43±0.02%), followed by leaves (0.31±0.01%), stem (0.21±0.06%), and was least in lateral roots (0.14±0.03%) and twigs (0.14±0.05%) (Fig 2K).

### Spatial distribution of metabolites in the seedlings of *D*. *binectariferum*

Based on the identities of the various ions assigned in the previous section, the spatial distribution patterns of the different ions in the seedlings (S1 Table) were analyzed using DESI MSI.

#### In cotyledons

**Fig 3:**
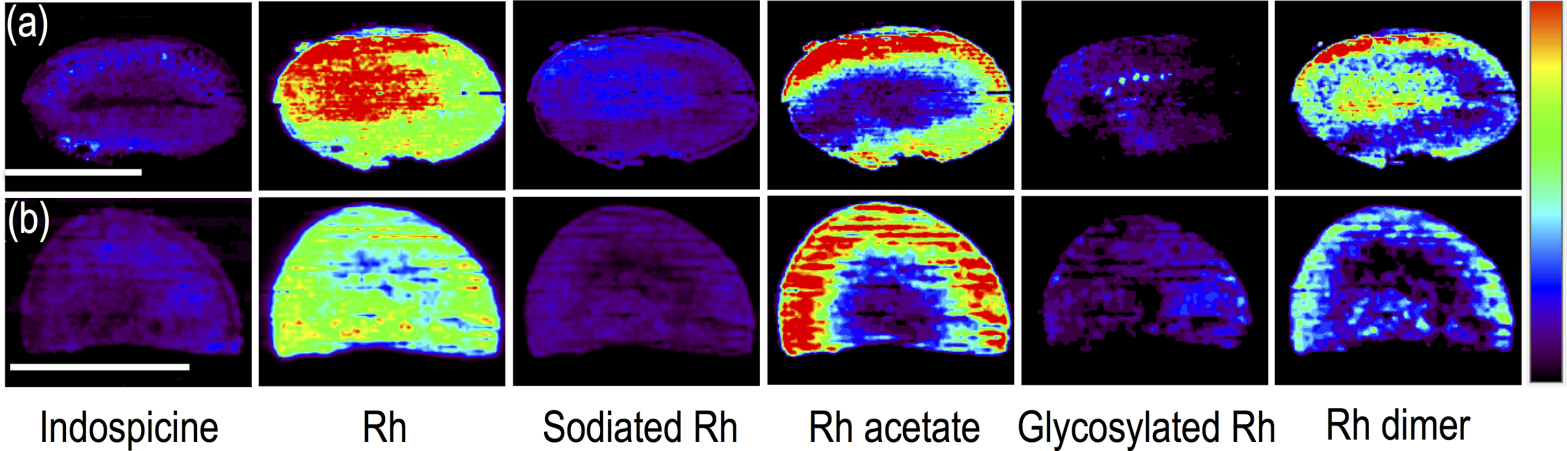
DESI MS images of (a) longitudinal and (b) cross section of cotyledons of *D*. *binectariferum*. Scale bars correspond to 5 mm and apply to all the images of a row. Intensity normalization of the images was done separately for all the individual metabolites.

DESI MS analysis of the cross and longitudinal sections of the cotyledon showed a few prominent metabolites (indospicine, Rh, sodiated Rh, Rh acetate, glycosylated Rh, and Rh dimer). All these, except indospicine are related to Rh. Though Rh (*m/z* 306.2) was uniformly distributed, the intensity was higher in the outer part of the cotyledon. This was also reflected in the distribution of acetylated Rh (*m/z* 348.2). The peak at *m/z* 915.4, possibly a trimer of Rh, reported in the seeds of *D*. *binectariferum* [26], was not recovered from the cotyledon (Fig 3).

#### In root and shoot

**Fig 4:**
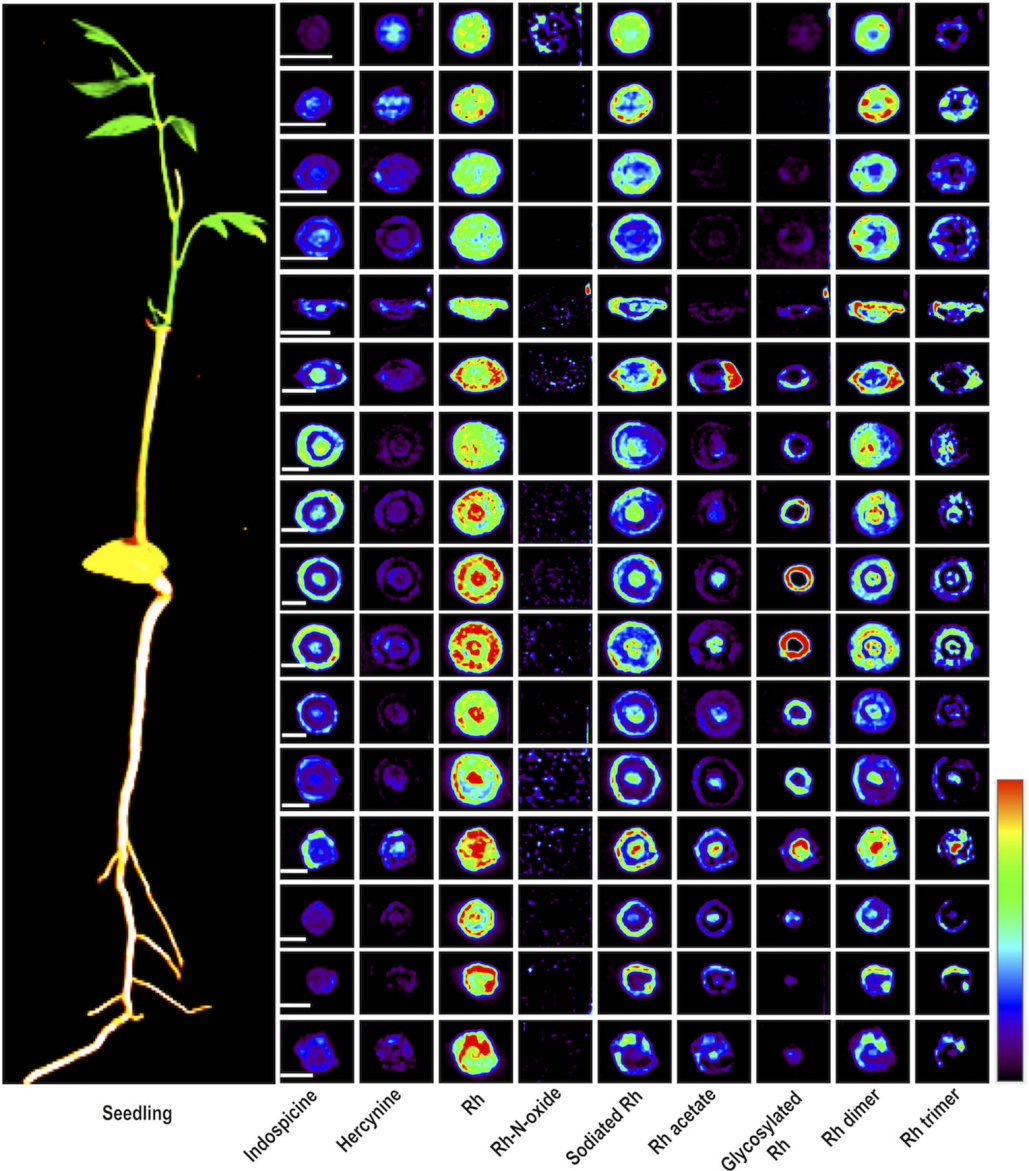
DESI MS images showing the distribution of Rh and other related compounds in different cross sections of root and shoot of a 10 months old seedling of *D*. *binectariferum*. Scale bars correspond to 2 mm and apply to all the images of a row. Intensity normalization of the images was done separately for all the individual metabolites.

Electron microscopic observation of cross section of the root clearly showed different parts corresponding to the epidermis, endodermis, cortex, xylem, and phloem tissues (Fig 1C and 1D). These observations helped in better visualization and description of the localization of metabolites in the tissue parts. DESI MS images of both the cross and longitudinal sections of the seedlings showed distinct stratification of the various ions in different parts of the seedlings. By far, the intensities of many of the ions were highest in the roots upwards to the collar region, representing the transition from the root to the shoot (Fig 4). Indospicine, hercynine, Rh, sodiated Rh, Rh dimer, and Rh trimer were present in all parts of the root and shoot while Rh acetate and glycosylated Rh were mostly present in the root and early development of shoot (Fig 4). Representative DESI MS spectra from a TLC imprint of cross sectioned root of *D*. *binectariferum* seedling are shown in S1 Fig.

Both longitudinal and cross section analyses indicated distinct tissue specific distribution of molecular ions. For example, indospicine, Rh, sodiated Rh, Rh dimer, and Rh trimer were found in cortex and endodermis, Rh acetate and glycosylated Rh in xylem and phloem regions, hercynine in endodermis, xylem, and phloem regions (Fig 4).

#### In leaves

**Fig 5:**
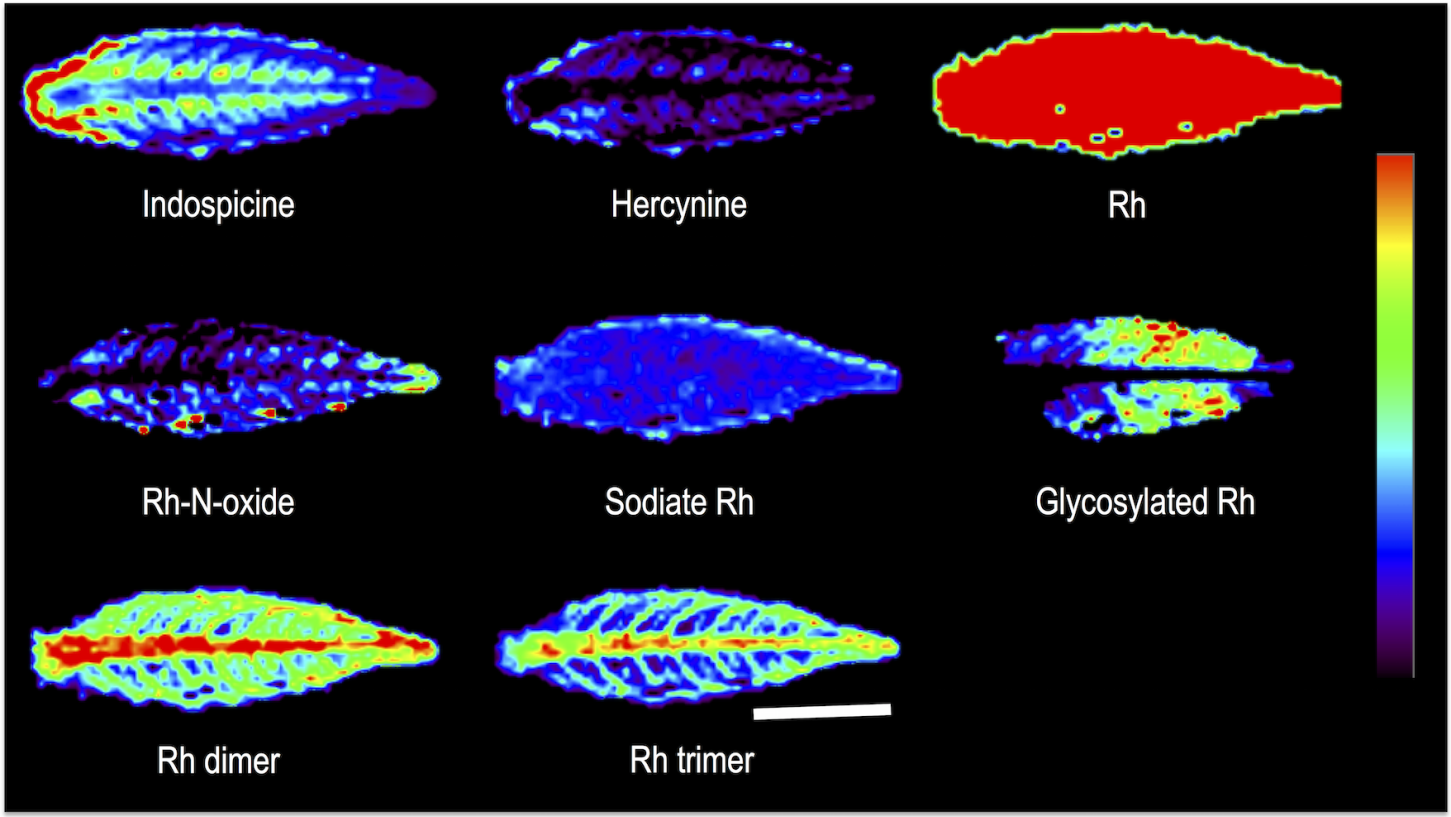
DESI MS images showing the distribution of Rh and other related compounds in the imprinted leaf of a 10 months old seedling of *D*. *binectariferum*. Scale bar corresponds to 5 mm applies to all the images.

Except Rh acetate, all other ions found in the root and stem were also present in the leaves (Fig 5). Many of the molecules detected in the leaves may be synthesized or accumulated in the leaves in response to various abiotic and biotic stresses. There was a distinct spatial pattern of the ions. For example, indospicine, Rh dimer, and Rh trimer were present in the midrib and veins, hercynine in leaf margin, Rh-N-oxide and glycosylated Rh in the leaf blade (Fig 5).

**Fig 6:**
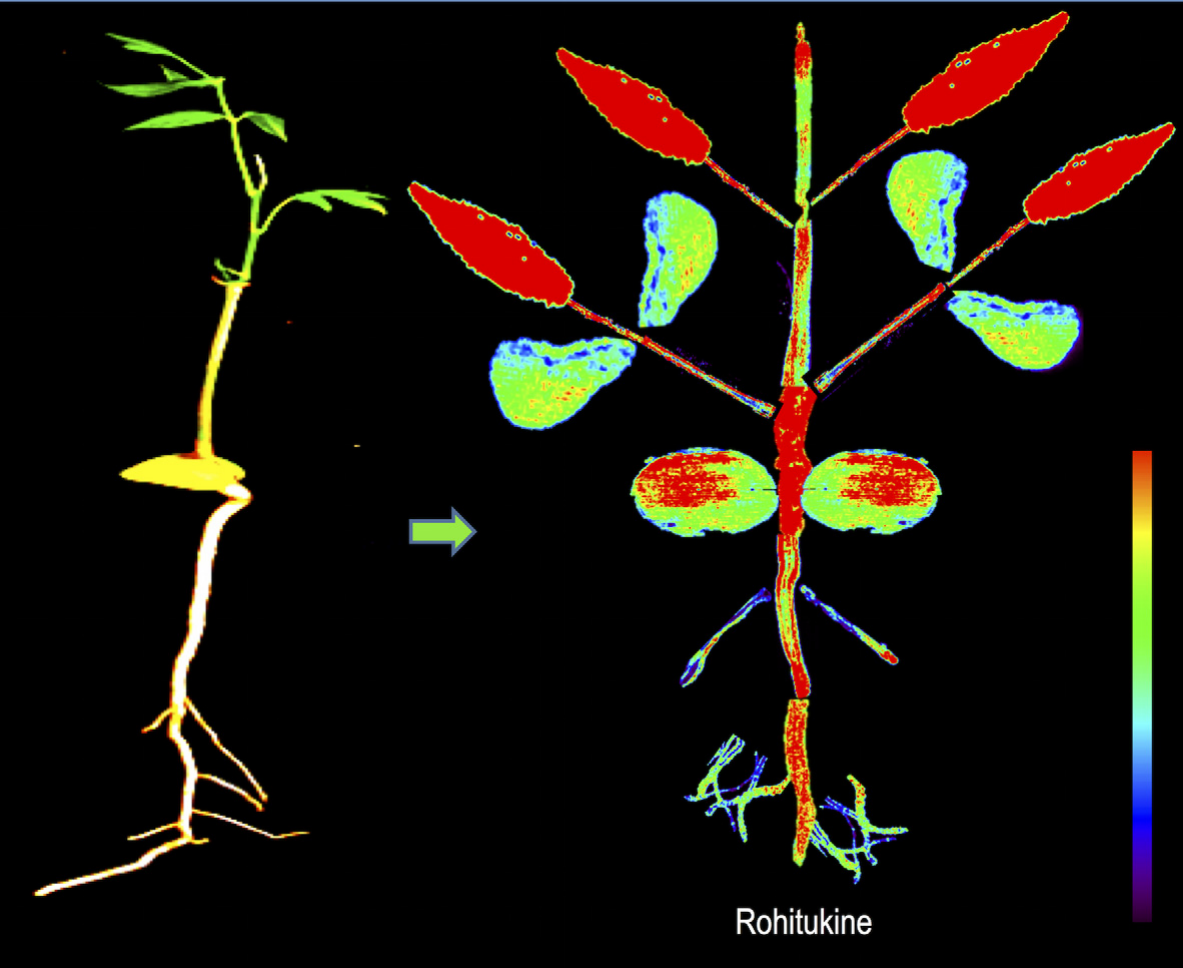
Reconstruction of spatial distribution of Rh obtained from DESI MS imaging of different parts of a 10 months old seedling of *D*. *binectariferum*.

Our study showed that Rh, one of the prominent chromone alkaloids in *D*. *binectariferum* seedlings, was predominantly distributed in the roots and leaves compared to the stem and twigs. Within the root itself, the distribution was restricted to the main roots and less in the secondary roots (Fig 6). In stems, Rh intensity was high in the collar region separating the root from the shoot (Fig 4). Finally, among the leaves, younger apical leaves were more densely packed with Rh than the older distal leaves (Fig 6).

In a recent study, we examined the spatial and temporal distribution of Rh and related compounds in the seeds of *D*. *binectariferum* during different seed developmental stages [26]. We showed that Rh was predominantly localized to the cotyledonary tissues with very little in the seed coat. Presumably such high levels of Rh in the cotyledonary tissues might act as a reservoir for translocation to the growing axis of the plant during seed germination.

While there is no unequivocal evidence, it appears that Rh could be synthesized both in the roots as well as in the growing shoot apices of the plant. Within the root or stem, Rh was clearly restricted to the cortex region in and around the phloem tissues indicating that it could be actively translocated through the phloem tissues from their sites of production in the plant (Fig 6). Additional support that there could be active transport of the metabolite comes from our finding of glycosylated Rh in different parts of the seedlings. Glycosylation of secondary metabolites has been reported in many instances [45–47]. Glycosylation leads to both stabilization and translocation of secondary metabolites through secondary transporters such as the ABC transporters. Furthermore, the glycosylation could also help in the storage of potentially toxic compound Rh until it is required upon to serve as a deterrent to pests [48, 49]. Though the functional significance of Rh in plants is not known, it has been shown to possess a variety of interesting pharmacological properties such as its ability to inhibit cyclin-dependent kinases CDKs [11]. It is likely that, this activity of Rh could serve as a defense for the plant against herbivore and hence explains the higher accumulation of Rh in the younger growing shoot apices.

**Fig 7:**
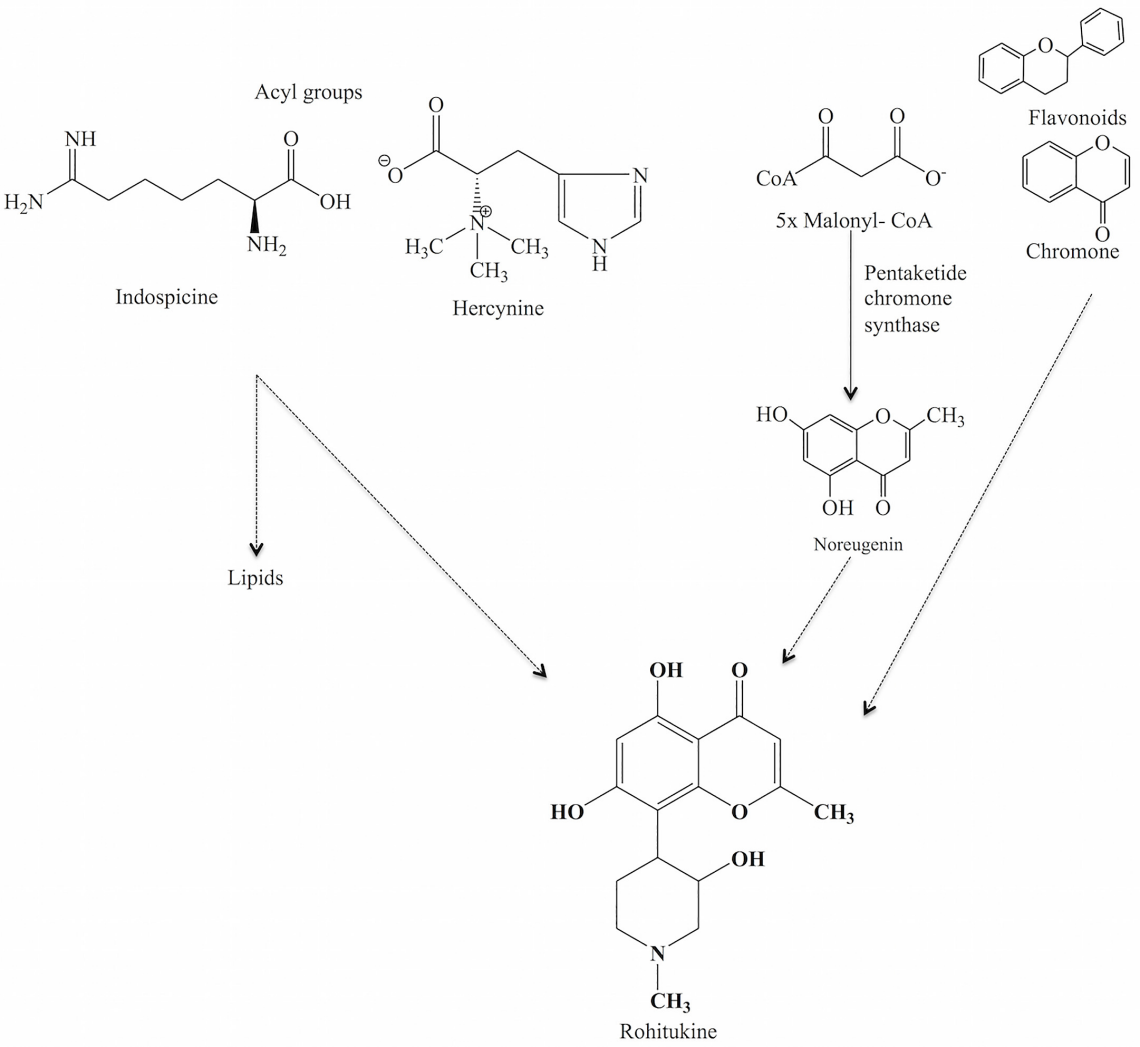
Schematic representation of possible biosynthetic pathway of Rh in *D*. *binectariferum*.

The biosynthesis of Rh is not yet elucidated. However, based on the constituent chemical moieties of the compound, it is suggested that it may comprise of the shikimic acid pathway to produce the flavonoids or the pentaketide pathway to produce chromone with a nitrogenous group derived from L-ornithine or other amino acids (Fig 7) [16, 20]. The biosynthesis of chromone alkaloid is hypothesized to follow either of the two major steps; a) involving noreugenin or b) involving a flavone compound (Fig 7) [3]. ESI MS and DESI MS data revealed that, besides Rh, a few other related chromone alkaloids, such as Rh-N-oxide, Rh acetate, glycosylated Rh, etc. were present in different parts of the plant. The detection of these chromone compounds strongly suggests the possibility of the involvement of the noreugenin pathway in the production of Rh in *D*. *binectariferum*.

In the recent past, a number of studies have used several imaging techniques including MALDI MS and DESI MS to unravel the spatial patterns in the distribution of plant secondary metabolites [28–33]. Understanding the spatial patterns could also lead to deciphering the underlying gene expression related to the pathway genes [34, 36]. In our study, younger leaves found to have higher levels of Rh compared to older leaves (Fig 7). It would be interesting to study the comparative transcriptomic profile of young and old leaves and unravel critical genes that are either expressed (in young leaves) or repressed (in older leaves).

## Conclusions

In conclusion, our results provide a spatially explicit distribution of metabolites in the seedlings of a tropical tree, *D*. *binectariferum*, with specific reference to a not-so-common group of metabolites, the chromone alkaloids. The mass spectrometric images provide a direct visualization of the localization of metabolites in the various parts of the seedlings. Considering a resolution of 250 μm, the images provide a sufficiently higher resolution display of the metabolites at a whole seedling level. Combination of these display data with transcriptomic data would provide a powerful way to unravel the genetic basis of the differences in the metabolite profiles. Currently work is underway in our laboratory to address this issue.

## Supporting Information

S1 Fig Representative DESI MS spectra and images of metabolites from the TLC imprint of cross sectioned root of *D*. *binectariferum* seedling. Scale bar corresponds to 2 mm applies to all the images.
(TIFF)

S1 Table Table showing list of *m/z* values of the ions, fragment ions, their probable chemical formulae, structures, and METLIN database ID. (PDF)
