# Supplementary figures and images for: Desorption Electrospray Ionization (DESI) Mass Spectrometric Imaging of the Distribution of Rohitukine in the Seedling of Dysoxylum binectariferum Hook. F
Source: PLoS One. 2016 Jun 30;11(6):e0158099. doi: 10.1371/journal.pone.0158099 (PMC4928942; doi:10.1371/journal.pone.0158099)

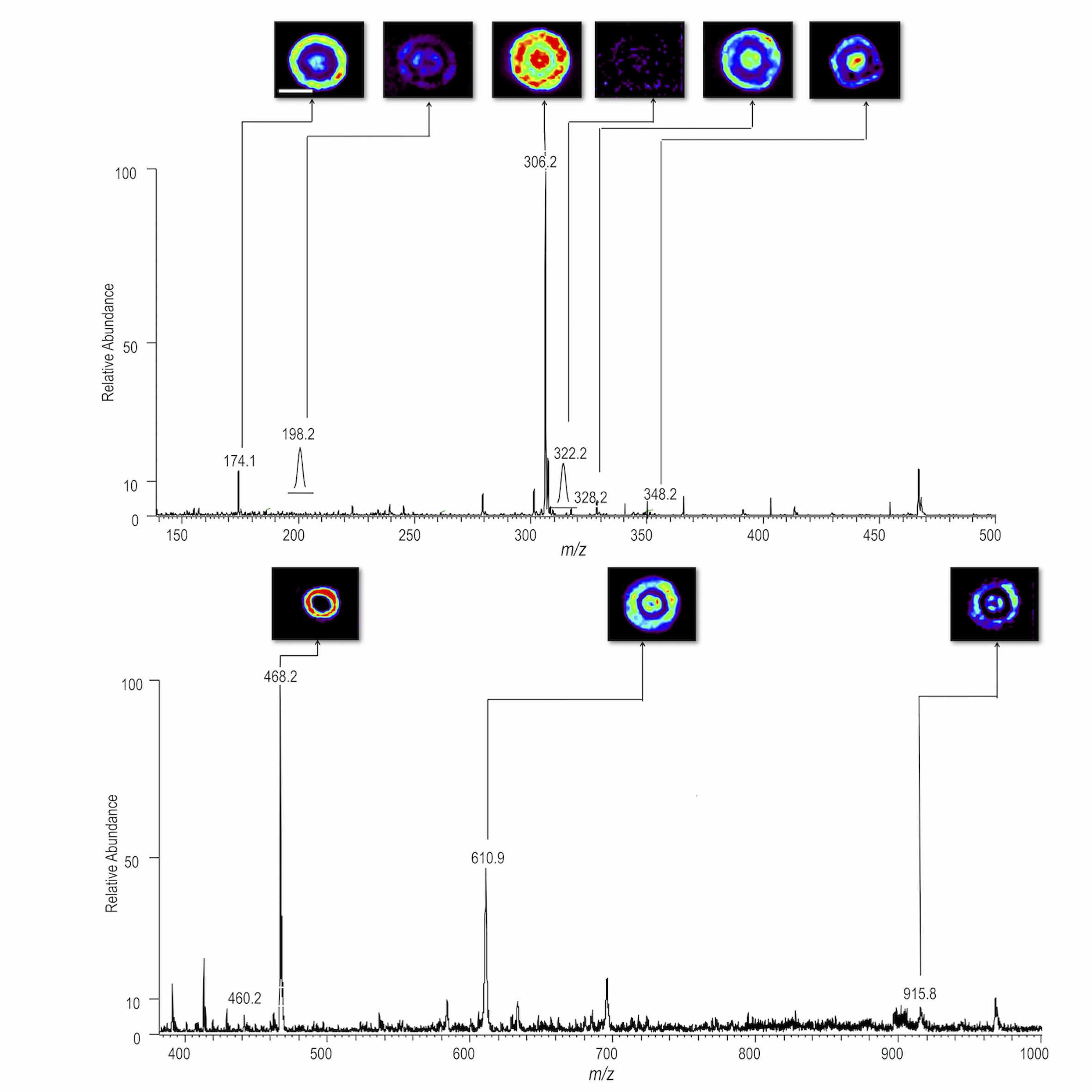

Supplement: S1 Fig — Scale bar corresponds to 2 mm applies to all the images. (TIFF) [file pone.0158099.s001.tiff]
