# Supplementary material for: Desorption Electrospray Ionization (DESI) Mass Spectrometric Imaging of the Distribution of Rohitukine in the Seedling of Dysoxylum binectariferum Hook. F
Source: PLoS One. 2016 Jun 30;11(6):e0158099. doi: 10.1371/journal.pone.0158099 (PMC4928942; doi:10.1371/journal.pone.0158099)
Supplement: S1 Table — (PDF) [file pone.0158099.s002.pdf]

Sl. no	*m/z* obtained from DESI MS	*m/z* obtained from ESI MS (Orbitrap)	Calculated exact mass of the ion	Probable metabolite name	Probable chemical formula of the ion	ESI MS/MS fragmented ions OR Class of metabolite	Structure	Tissue where metabolite localized	METLIN-ID
1	174.1	174.1236	174.1237	Indospicine	$C_7H_{15}N_3O_2$+H	156.1, 116.1	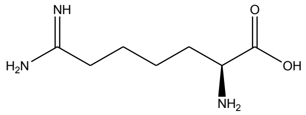	Cotyledon<br>Cortex and endodermis, midrib and leaf veins	3299
2	198.2	198.1235	198.1237	Hercynine	$C_9H_{15}N_3O_2$+H	180.0, 154.0, 95.0	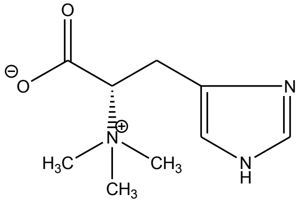	Endodermis, xylem, and phloem<br>Leaf margin	63493

3	306.2	306.1336	306.1335	Rohitukine	$C_{16}H_{19}NO_5$+H	288.0, 245.0, 222.0	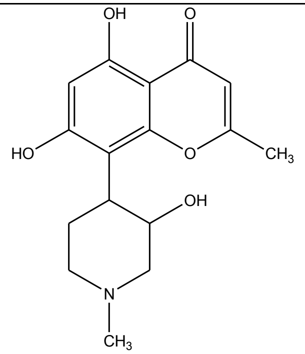	Cotyledon<br>Cortex and endodermis<br>Leaf	
4	322.2	322.1283	322.1285	Rohitukine-N-oxide	$C_{16}H_{19}NO_6$+H	304.3, 276.2, 238.0	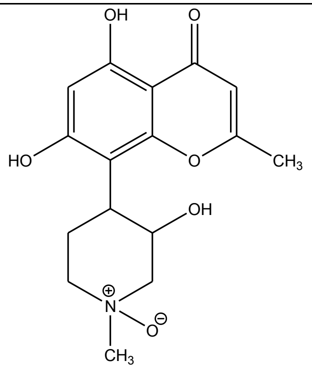	Leaf blade	
5	328.2	328.1155	328.1155	Rohitukine	$C_{16}H_{19}NO_5$+Na	-	-	Cotyledon<br>Cortex and endodermis<br>Leaf	

6	338.2	338.1599	338.1598	Methoxylated rohitukine analogue	$C_{17}H_{23}NO_6$+H	306.2 and 288.0, 245.0, 222.0	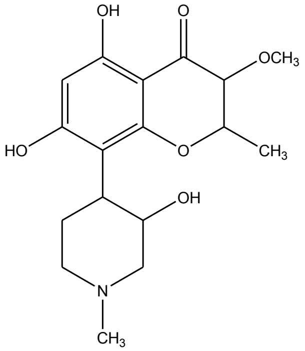	Leaf	
7	348.2	348.1447	348.1442	Rohitukine acetate	$C_{18}H_{21}NO_6$+H	306.2, 288.0, 245.0	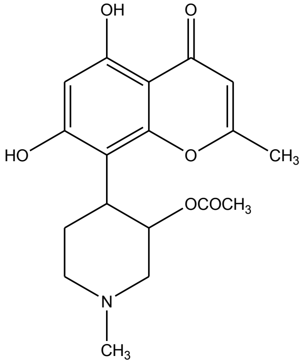	Cotyledon<br>Xylem and phloem	

8	401.1	401.2083	401.2087	17beta-Hydroxyestr-4-en-3-one benzoate	$C_{25}H_{30}O_3$+Na	Steroid ester	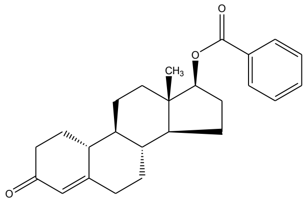	Epidermis, cortex, and endodermis	70661
9	468.2	468.1865	468.1864	Glycosylated rohitukine	$C_{22}H_{29}NO_{10}$+H	306.2 and 288.0, 245.0, 222.0	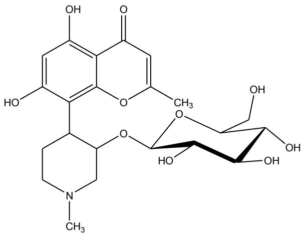	Cotyledon<br>Xylem and phloem<br>Leaf blade	
10	490.2	490.1687	490.1684	Bis-N-butyl phthalate	$C_{22}H_{29}NO_{10}$+Na	Carbohydrates	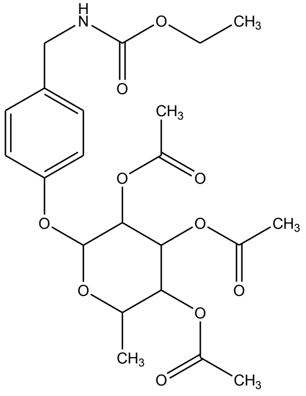	Xylem and phloem	88062
11	610.9	611.2598	611.2600	Protonated rohitukine dimer	$C_{32}H_{38}N_2O_{10}$+H	-	-	Cotyledon<br>Cortex and	

								endodermis, midrib and leaf veins	
12	527.3	527.1583	527.1583	Maltotriose	$C_{18}H_{32}O_{16}$+Na	365.1 and 348.0, 204.0, 185.2	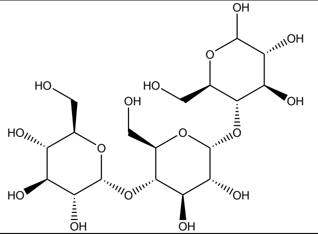	Leaf blade	3585
13	649.2	649.2127	649.2127	Embigenin 2''-(2'''-acetylrhamnoside)	$C_{31}H_{36}O_{15}$+H	Flavonoids	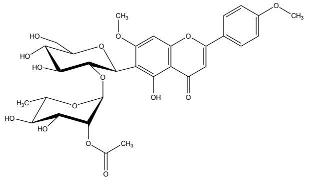	Midrib and leaf veins	49452
